# Photonic Design and Electrical Evaluation of Dual-Functional Solar Cells for Energy Conversion and Display Applications

**DOI:** 10.1186/s11671-019-2901-6

**Published:** 2019-02-28

**Authors:** Jun Du, Yidan An, Cheng Zhang, Canyan Zhu, Xiaofeng Li, Dong Ma

**Affiliations:** 1 School of Rail Transportation, Soochow University, Suzhou, 215131 China; 2 School of Optoelectronic Science and Engineering and Collaborative Innovation, Center of Suzhou Nano Science and Technology, Soochow University, Suzhou, 215006 China; 3 Key Lab of Advanced Optical Manufacturing Technologies of Jiangsu Province and Key Lab of Modern Optical Technologies of Education Ministry of China, Soochow University, Suzhou, 215006 China

**Keywords:** Colored solar cells, Esthetic building-integrated photovoltaics, RGB imaging applications, Optoelectronic simulation

## Abstract

Colored solar cells (SCs) are highly useful for applications in esthetic building-integrated photovoltaics (BIPVs). However, the theoretical designs mostly focus on the color quality with rarely addressing the optoelectronic responses. Here, considering both color display and complete electrical evaluation, we report a color-controlled a-Si:H SC in purely planar configuration, which simultaneously exhibits the desired high-purity color and sustains a relatively high power conversion efficiency. The high-performance color display is realized by thin-film photonic designs with incorporating distributed Bragg reflector and anti-reflection coating layers. Moreover, a comprehensive optoelectronic simulation addressing both the electromagnetic and internal semiconductor physics has been realized, which shows that the power conversion efficiencies of the designed red-green-blue (RGB) SCs can be 4.88%, 5.58%, and 6.54%, respectively. The physical principles of optimizing the colorful SCs with the tunable hue, high saturation, and brightness are explained, and we take the logo of “Soochow University” as an example to demonstrate the wide-angle pattern display by the SCs. The study paves the way of realizing the colored SCs targeting esthetic BIPV applications.

## Background

In the wake of the global energy crisis and extensive urbanization, significant efforts have been devoted into the building-integrated photovoltaics (BIPVs). Particular attention has been paid for the next-generation (zero-energy) buildings with the electricity consumption equivalent to the generation [1–5]. Unfortunately, the conventional photovoltaic devices show dull or black colors and hence the BIPV based on such solar cells (SCs) cannot meet the requirement of esthetic sense [6]. Recently, the color-controlled SCs with the benefits of displaying various colors and vivid patterns (besides their electricity-generation functionalities) are attracting increasing interests due to their huge market prospects [7, 8].

On the one hand, various photonic approaches can be used to control the optical responses of the SCs to show specific colors, including (1) employing a Fabry-Perot (F-P) filter upon the SCs to control the color and purity by tailoring the F-P resonance [9–11] and (2) incorporating a color-adjusting layer (CAL) above (or behind) the transparent conducting oxide (TCO) layer or replacing the TCO by CAL completely. For example, selectively transparent and conducting photonic crystal (STCPC) can be used as the rear contact to control the transmission spectrum and the color through the BIPV devices [12, 13]; the distributed Bragg reflector (DBR) can be integrated to display colors of the thin-film SCs and organic photovoltaics [14, 15]. Despite most of those literature focus on obtaining the color display and electricity output simultaneously, the color purity is lower and the color space is insufficient for pattern displays. What is more, these methods sacrifice too much energy conversion efficiency of SC in order to achieve color display. Colorful SCs with higher color purities are of high significance for the development of BIPV technology.

On the other hand, theoretical literature focus preferentially on the optical design of the SCs in order to display various colors [6, 8, 16]; however without strictly examining the intrinsic carrier behaviors inside the device. For the design of SCs, it is highly necessary to investigate how the special optical design modifies the carrier generation, transport, and collection processes within the semiconductor junctions, which play the key roles in determining the operation and performance of SCs [17–19]. However, a comprehensive device-level simulation for highly nanostructured SCs is challenging since the concerned devices show very complicated multi-domain behaviors, e.g., with very rich optical resonances and carrier generation/recombination/collection responses which show strong dependences on the space, wavelength, and many other ingredients [20–22]. Further, since the fabrications of such specific SCs are always time-consuming and costly, a comprehensive design of the colorful SCs by addressing the photonic as well as internal carrier responses is highly beneficial for the development of this kind of solar device.

In this article, we present a complete optoelectronic study on the color-controlled a-Si:H SCs. Optically, to realize the high-purity red-green-blue (RGB) display, we introduce the DBR as the color-selective component and the additional dual-layer antireflection coatings (ARCs) as the color-optimization component. It shows that the obtained color space from this study can be comparable to that of the standard RGB (sRGB) system. Electrically, the intrinsic generation, transport, recombination, and collection of electrons and holes within the designed RGB a-Si:H SCs are addressed so that a complete list of the photoconversion performances of the SC can be achieved. By evaluating the external-quantum efficiency (EQE) spectra and current-voltage (*J*-*V*) characteristics, we find that the power conversion efficiencies of the SCs with high-purity red, green, and blue colors are 4.88%, 5.58%, and 6.54%, respectively. Finally, to demonstrate the possibility of RGB imaging, the logo of “Soochow University” is designed and realized by using the color-controlled a-Si:H SCs; the pattern displayed is well sustained under a large range of incident angle.

## Methods

The optical response is calculated by solving Maxwell’s equations via the rigorous coupled-wave analysis (RCWA) and COMSOL Multiphysics. The reflection, absorption by each layer, etc., can all be obtained. The detailed electrical characteristics (e.g., carrier generation/recombination/collection) are obtained by the electromagnetic and carrier transport calculation, as introduced detailedly in our previous papers [17–22]. The optical reflection spectrum could be transformed into related parameters in CIE color system, then the resulting color sample could be obtained through CIE chromaticity coordinates. The calculation of this transform follows a series of chromaticity standards developed by CIE. The thickness of ZnS and ZnO are fixed based on the thin-film optics law and the thickness of SC is fixed at 500 nm. The complex refractive coefficient of materials is taken from the Palik [23]. A mesh size of 5 nm was used in the simulated region, and perfectly matched layers were employed at the boundary condition for the optical simulation. For the electrical simulation, the Poisson equation and the carrier transport equations are obtained, in which the surface recombination and metal contact are chosen as the boundary situations.

## Results and Discussion

**Fig. 1:**
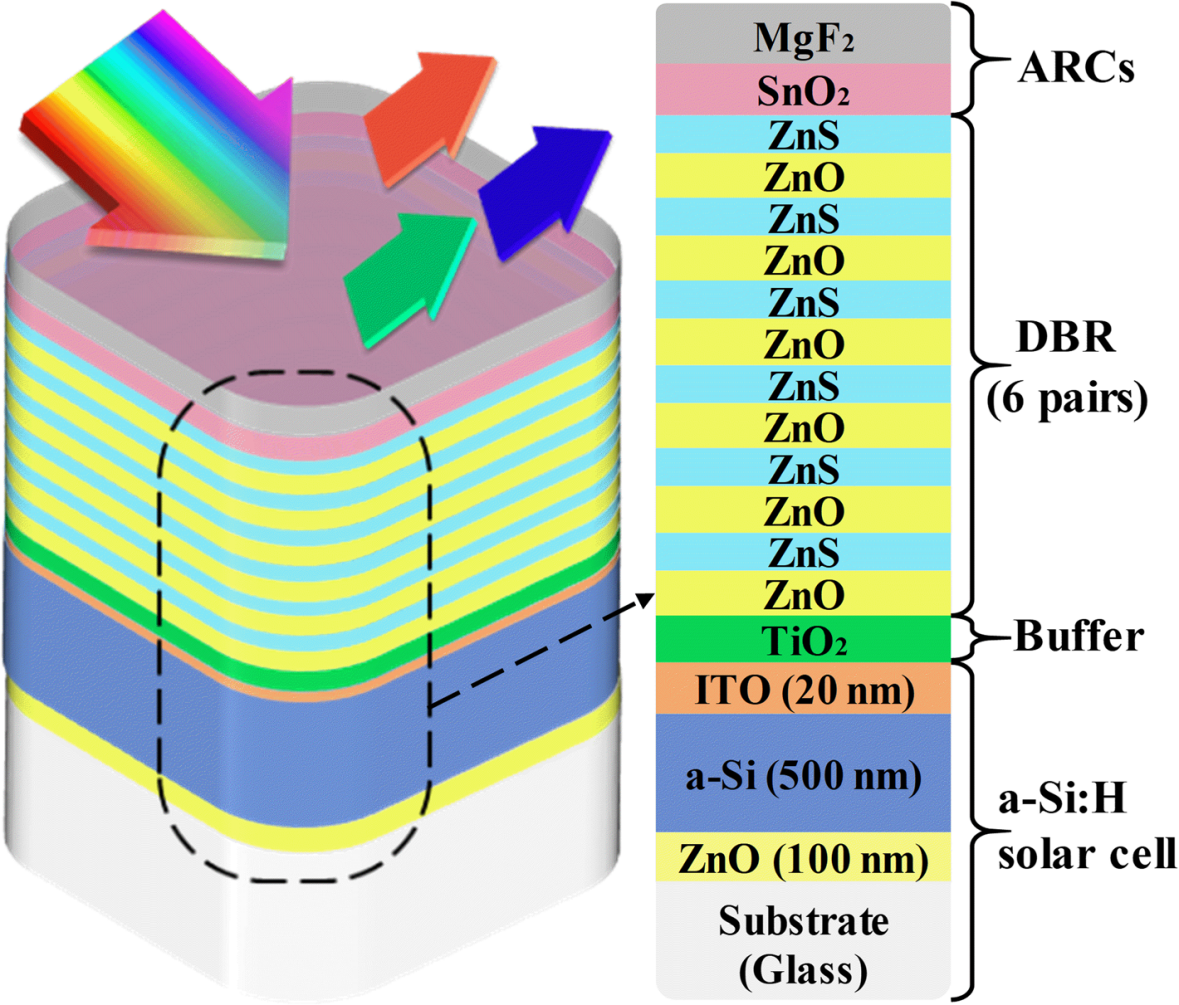
Schematic diagram of the proposed color-controlled a-Si:H SCs (left) and the detailed device configuration (right)

Shown in Fig. 1 is the schematic diagram of the proposed color-controlled a-Si:H SC. From top to bottom, it consists of ARC layers, DBR stack, buffer layer, and the a-Si:H SC. Here, the thickness of the a-Si:H active layer is 500 nm which contains 30 nm (50 nm) n-type (p-type) doping zone. The material of the rear (front) electrode for electron (hole) transport is ZnO (ITO) with a thickness of 100 nm (20 nm). The buffer layer is composed of 55 nm TiO_2_ to reduce the light reflection [24] and improve the color purity. The DBR is composed of 6 ZnS/ZnO pairs with the quarter-wavelength thickness for each layer. In fact, the reflectivity and spectral width play very important roles in determining the color quality. The reflectivity (*R*) of DBR can be analytically predicated by using the following equation [25]:

1 $$ R={\left[\frac{n_0{\left({n}_2\right)}^{2N}-{n}_s{\left({n}_1\right)}^{2N}}{n_0{\left({n}_2\right)}^{2N}+{n}_s{\left({n}_1\right)}^{2N}}\right]}^2 $$

where *n*_0_, *n*_1_, *n*_2_, and *n*_s_ are the refractive indices of air, the two DBR layers, and the substrate, respectively; *N* is the number of DBR pairs. The reflectance bandwidth (∆λ_0_) is [25]:

2 $$ \Delta \lambda =\frac{4{\lambda}_0}{\pi } arc\sin \left(\frac{n_2-{n}_1}{n_2+{n}_1}\right) $$

where λ_0_ is the DBR central wavelength. It is noted that increasing the difference of *n*_1_ and *n*_2_, *R* is getting higher (i.e., the increased color brightness), but ∆λ_0_ and color saturation are decreased. In consequence, a relatively small difference of *n*_1_ and *n*_2_ together with a relatively large *N* is used to ensure a high saturation to present the high color purity and brightness.

**Fig. 2:**
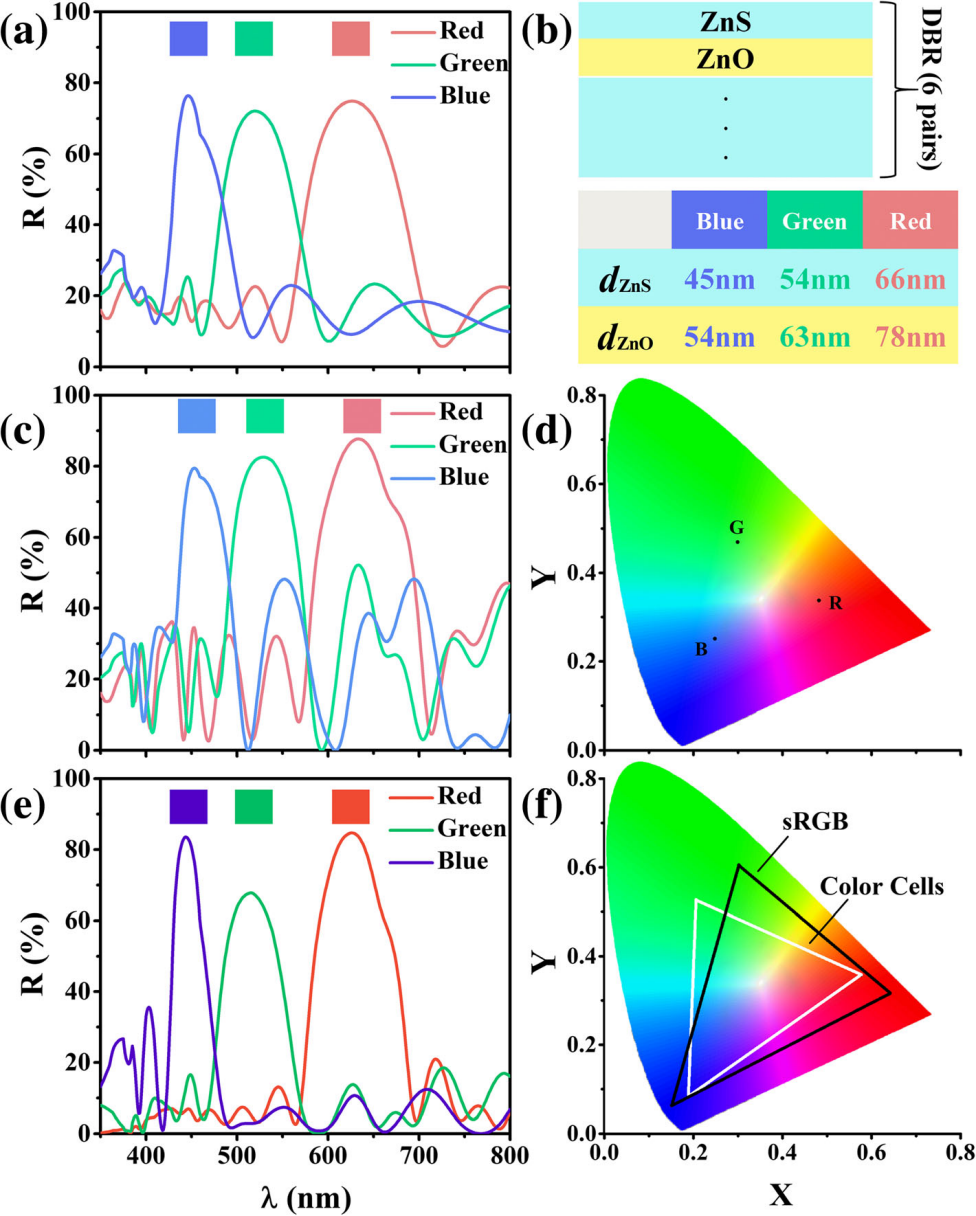
Optical responses of DBRs and RGB a-Si:H SCs. **a** DBR reflection spectra targeting RGB display. **b** Structural and material parameters of the designed DBR. Reflectivity spectra (**c**) and the CIE 1931 chromaticity coordinates (**d**) of the a-Si:H SCs with RGB DBRs atop. Reflectance spectra (**e**) and the CIE 1931 chromaticity coordinates (**f**) of the designed color-controlled a-Si:H SCs. The standard sRGB color gamut is inserted in (**f**) for comparison

According to the thin-film optics, the DBR thicknesses have to be carefully designed in order to display the RGB colors localized differently in the visible band. Here, excluding the SCs, we first examine the controllability of the DBR reflection spectrum for RGB display. Figure 2a shows the reflection spectra of DBRs under RGB designs, with the corresponding structure and film thicknesses given in Fig. 2b. It is found that the reflections are peaked at λ_0_ = 625, 520, and 445 nm, respectively, which well match the RGB centers. Moreover, the peaked reflections are strong enough (i.e., 74.82%, 72.1%, and 76.31%) to ensure the displaying brightness. In fact, for DBR, there exist some side waves out of the forbidden band. Such waves are detrimental for achieving the high color purity [26]. Figure 2a verifies the existence of such side waves.

Above RGB, DBRs are now integrated with the a-Si:H SCs, i.e., RGB-DBR (top) + SC (bottom). The reflection spectra of the combined SC systems targeting the RGB display are shown in Fig. 2c. It is first observed that the central wavelengths with incorporating the SC have been slightly red-shifted (from 625, 520, and 445 nm to 633, 528, and 453 nm for R, G, and B cells, respectively); moreover, the peak reflections are also increased to 87.66%, 82.52%, and 79.44%, respectively. This is reasonable since the inclusion of the SC beneath the DBR has changed the system configuration and modified the resonant situation. Despite so, the above effect is relatively weak without affecting the displaying quality. However, there is indeed a key ingredient which strongly degrades the color purity, i.e., the much intensive side waves arising from the increased reflectances at the interfaces of the SC. Figure 2d depicts the Commission Internationale de L’Eclairage (CIE) 1931 chromaticity coordinates for these combined SC systems. For pattern display applications, the larger the color space, the more color elements it contains, and the better it displays [27]. When the primary colors are closed to the tongue-shaped boundary, the largest color space can be obtained. However, Fig. 2d shows that the achieved RGB are relatively far from the boundary; therefore, we need to further decrease the reflection bandwidth as well as eliminate the side waves.

To improve the RGB performance, we further introduce the dual-layer ARCs (MgF_2_ and SnO_2_) together with a buffer layer (TiO_2_). The ARCs are configured on top of DBR and the buffer layer is sandwiched by DBR and a-Si:H SC as shown in Fig. 1. Based on thin-film optics, the thickness of the ARCs can be controlled by [28]:

3 $$ {n}_t^2={n}_b^2\cdot \frac{n_0}{n_s}\mathrm{and}\ {d}_t=\frac{\lambda_0}{4{n}_t};\kern0.5em {d}_b=\frac{\lambda_0}{4{n}_b} $$

where *n*_0_, *n*_t_, *n*_b_, and *n*_s_ are the refractive indices of the air, top layer, bottom layer, and substrate, respectively; *d*_t_ and *d*_b_ are the thicknesses of the top and bottom layers, respectively. Plotted in Fig. 2e are the reflection spectra of the designed color-controlled a-Si:H SCs with ARCs, DBR, and buffer layer. It is distinct that (1) the peak wavelengths are 625, 515, and 445 nm, close to those from the stand-alone DBRs; (2) the resonant bandwidths are strongly decreased for the cell with RGB colors; (3) the side waves are dramatically suppressed, even compared with the results of DBRs alone shown in Fig. 2a. As expected, after introducing the ARCs and buffer layers, the optical path differences have been changed, varying the resonant situation. As a result, the central wavelengths, reflection bandwidth, and the side waves of system are improved. Therefore, the advanced photonic designs lead to the desired color with much-promoted color quality as proved by the CIE 1931 chromaticity coordinates in Fig. 2f. Compared to the sRGB, the color differences between the designed RGB and sRGB are as follows: ΔE_R_ = 16.8 for red, ΔE_G_ = 47.6 for green, and ΔE_B_ = 41.7 for blue. Despite the color differences show a slight change between the designed RGB and sRGB from the observer perceives, the color space for our design is comparable to that of the sRGB. For example, the RGB color spaces are approximately equal to 52.7% (72%) of National Television System Commission (NTSC) color spaces for the designed and standard systems, respectively.

Up to now, we have successfully designed the a-Si:H SCs with an advanced thin-film optical strategy. However, for such a displaying function, the electrical response of the SCs will be affected inevitably. Therefore, it is of necessity to examine the detailed optoelectronic response of the color-controlled a-Si:H SCs. In the past years, we have performed extensive studies on the device-level simulations of semiconductor-based SCs, including optoelectronic simulation with addressing the electromagnetic and carrier transport responses [17, 18] as well as the advanced opto-electrical-thermal simulation of SCs [19]. SCs based on (1) various materials (e.g., Si, GaAs, and a-Si:H) and (2) various nanostructures (e.g., single-nanowire, nanotextures, and double-junctions) have been explored in order to find the ways of controlling the intrinsic multiphysics behaviors inside the SCs and improving the photoconversion efficiencies [20–22]. Therefore, the optoelectronic response of the specially designed a-Si:H SCs presented in this paper can be readily obtained by performing the corresponding optoelectronic simulation.

**Fig. 3:**
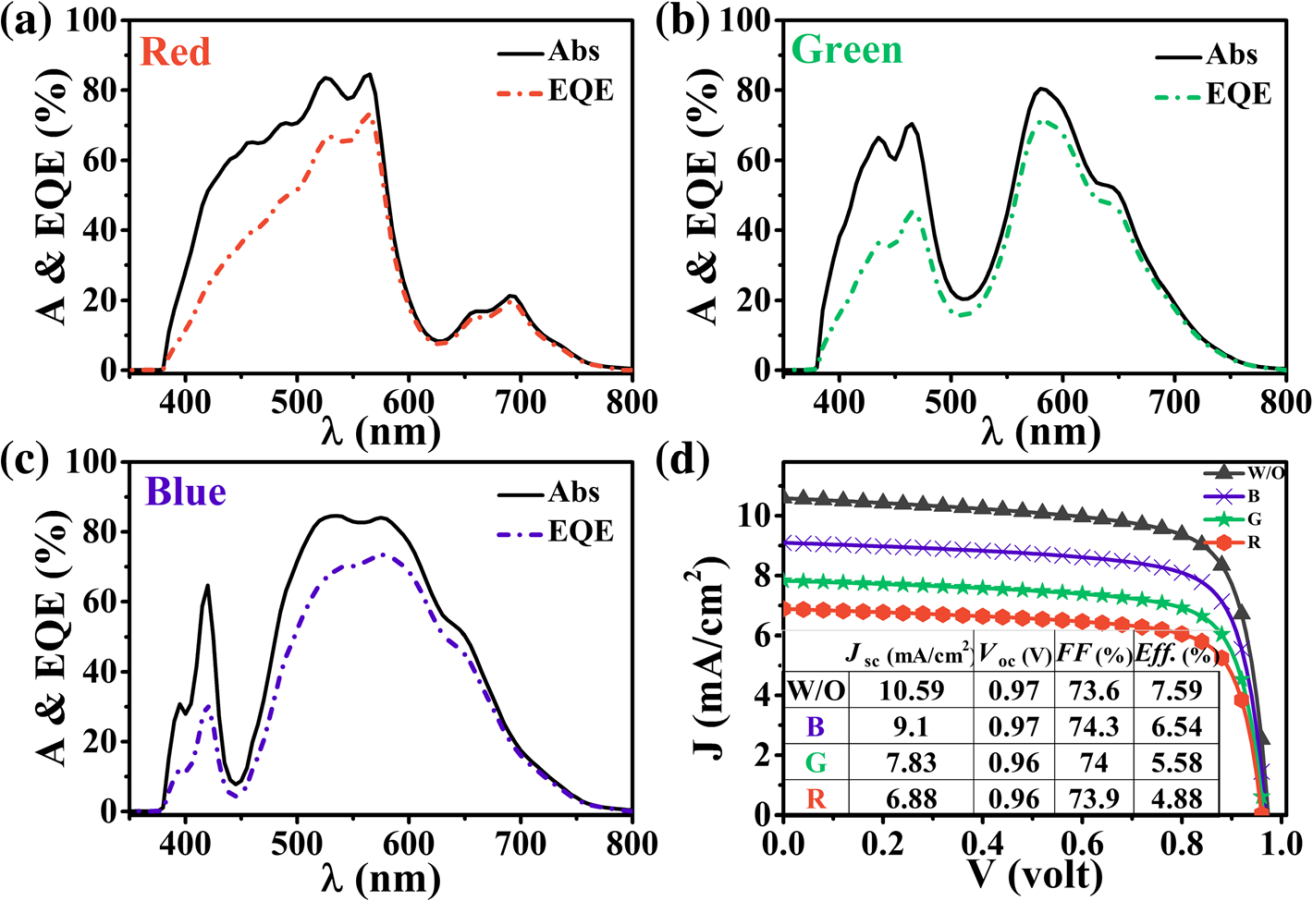
Absorption and EQE spectra of the color-controlled a-Si:H SCs with color of **a** red, **b** green, and **c** blue. **d** IV curves of the designed a-Si:H SCs, where the original system without RGB design is included for reference. The inserted table shows the *J*_SC_, *V*_OC_, FF, and Eff

Figure 3a–c shows the absorption (*A*) and EQE spectra of the RGB SCs under AM1.5 illumination. First, it is shown that the absorption spectra show apparent dips at specific wavelengths corresponding to the reflection peaks for R, G, and B colors, respectively. This is because the color-displaying function requires the specific light reflections at the visible band; thus, the optical absorption (*A*) and electrical response (EQE) of the SCs will be affected inevitably, leading to a substantial difference in EQE and *A* for red, green, and blue SCs. Besides, at the wavelength band of less than 380 nm, we can see that the light is almost totally absorbed by the top ITO layer; hence, the corresponding absorption and EQE are close to zero. Despite so, the overall device absorption is good enough to show the peaked *A* over 80%. Second, since a-Si:H SC is considered in this study, the carrier recombination effect exists almost in the whole valid spectral band (because the active layer is very thin) so that the EQE is always lower than *A*. The corresponding current-voltage characteristics (*J*-*V* curves) are plotted in Fig. 3d, where the inset shows the detailed short-circuit current density (*J*_SC_), open-circuit voltage (*V*_OC_), fill factor (FF), and photoconversion efficiency (Eff) for the RGB cells. For comparison, a conventional a-Si:H SCs with a 100 nm SiO_2_ anti-reflection layer is used and shows an efficiency ~ 7.59%, which is similar to the report by Anderson et al. [16, 29]. It is found that the RGB design does not obviously affect the *V*oc and FF. It is acknowledged that the *V*_OC_ and FF of the SC are mainly determined by the intrinsic properties of material (e.g., band-gap), doping concentration of the active layer, and device configuration; hence, the RGB design affects the absorption, rather than the *V*_OC_ and FF. As expected, the color SCs show the decreased efficiencies due to the color-displaying purpose. More detailedly, the blue-colored SC has the maximum efficiency of 6.54%, while the green 5.58% and the red 4.88%. The red cell shows the largest efficiency reduction since the reflected red light has the strongest solar energy. This is a reasonable sacrifice for such a multi-functional SC.

It should be noted that, if we would like to further increase the energy conversion efficiency, more complex structure could be introduced. Optically, for instance, (1) the light-trapping effect (e.g., the TCO with textured surface) can be used; (2) the TCO surface can be covered with TiO_2_-ZnO antireflection layers (e.g., improve the quantum efficiency ~ 10% at 550 nm) [30]. Electrically, (1) a triode plasma-enhanced chemical vapor deposition (PECVD) technique can be used to depress the light-induced degradation effect [31]; (2) our optoelectronic simulation can optimize the carrier transport dynamic behaviors to further depress the carrier recombination and enhance the electricity output [18]. Moreover, this design principle is also applicable to other kinds of SCs (e.g., Perovskite, crystalline Si, organic, and hybrid SCs) [32]. Therefore, the energy conversion efficiency of the designed colorful SC could be increased by various photonic or electrical means.

**Fig. 4:**
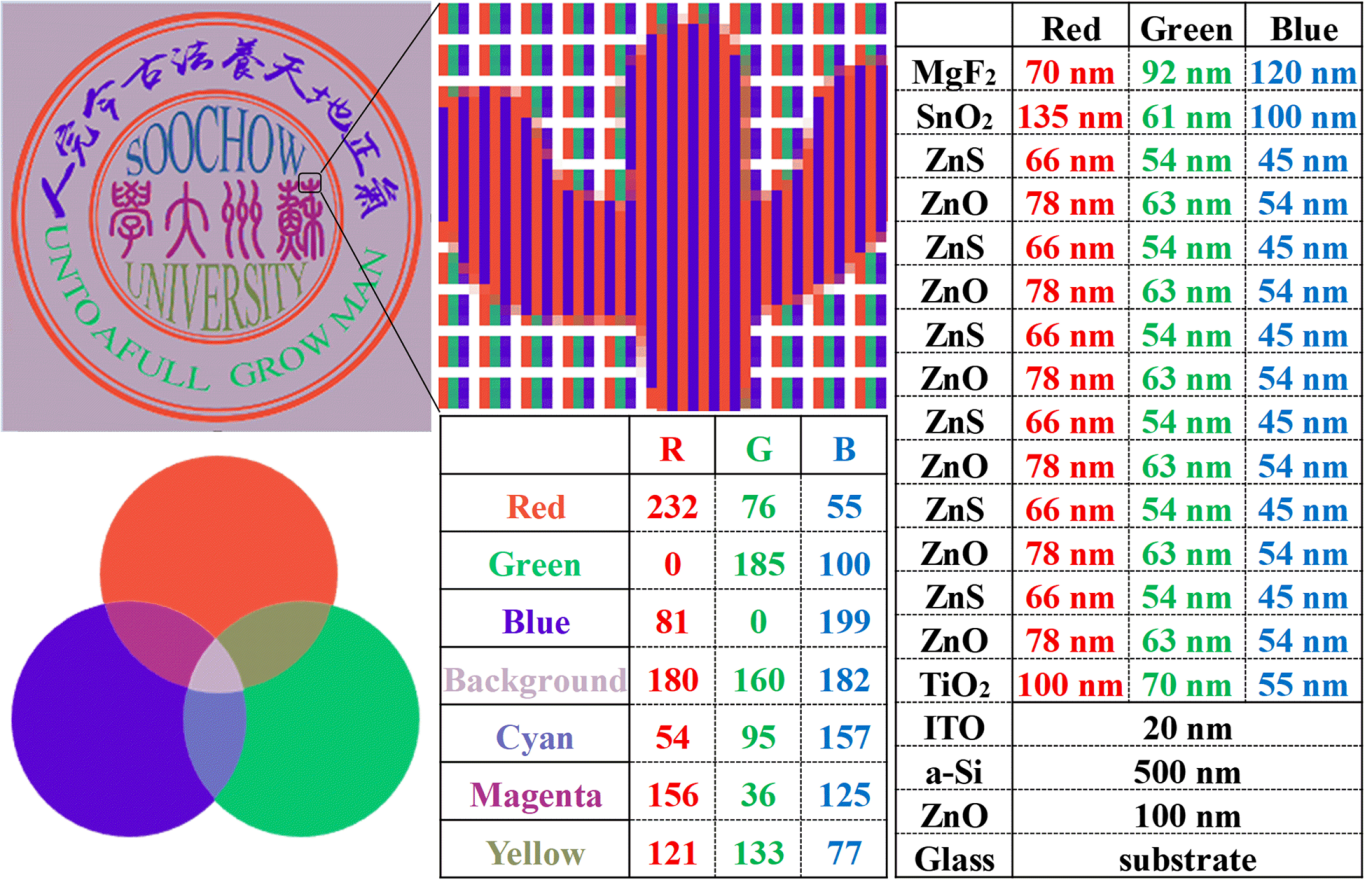
Logo of Soochow University, with pixels composed of RGB a-Si:H SCs. The inset shows the microscopic pixel composition, the structure details of the RGB a-Si:H SCs, and the RGB values of color maps mixed by the three primary colors

Next, we demonstrate the application of the a-Si:H SCs in pattern display and esthetic architecture. Figure 4 shows the designed logo of Soochow University (left top), the enlarged part of the logo (middle top), the detailed structure information for RGB designs (right), and the corresponding RGB values of seven colors in the logo (middle bottom). (1) There are seven color elements in the logo comprised from the primary RGB elements. (2) The four circles are red, the vocabularies bottom of outer ring are green, and the Chinese characters top of outer ring are blue directly from the color-controlled SCs. (3) The background exhibits a color of purple in gray, consisting of equal RGB contributions. The RGB value in the logo represents the three components of red, green, and blue. For example, for red, the smaller the values of green and blue, the larger the color saturation [33]. Therefore, the saturation of red and blue colors is higher than that of green, leading to the larger number of R and B than G values in the mixing color and makes it to be purple [34]. (4) The RGB values are not large enough compared to the maximum value of 255, leading to a low brightness and gray color. The central Chinese characters are magenta, consisting of equal red and blue, as shown by the enlarged illustration on the right-top of Fig. 4. (5) The chromatic aberration of magenta is smaller than the other mixing colors due to the better proportion of RGB components. The “SOOCHOW” (UNIVERSITY) are cyan (yellow), consisting of green and blue (red and green), respectively. Both have problems of RGB proportional imbalance and low brightness. Although there has the space for further improvement, the pattern is clear and distinguishable as a whole.

In practical applications, unlike the nanostructured SCs, the proposed RGB SCs in planar configuration can be fabricated by the very mature commercial fabrication processes [35]. At the bottom, there is a representative a-Si:H solar cell with a p-i-n structure. Firstly, the n-type amorphous silicon (n-a-Si:H) layer is deposited on a TCO-coated substrate (glass or plastic) by PECVD, intrinsic amorphous silicon (i-a-Si:H), and p-type amorphous silicon (p-a-Si:H) layers are followed by the same method. Then, the top electrode is usually the TCO layer, which is deposited by sputtering [36]. Next, the buffer layer is deposited on the complete a-Si:H SC, followed by the alternating layer of DBR using magnetron sputtering [37]. Eventually, RGB a-Si:H SCs is completed by depositing the top dual-layer ARCs with magnetron sputtering. In the preparation process, a variation of the thickness from 1 to 5% is possible. Therefore, in order to investigate the effect of the variation of the thicknesses, we introduce a random variation of the thickness (e.g., from − 5% to 5%) for each layer. The simulation results show that the color differences (ΔE) range from 1.9 to 11.2 for red, 1.3 to 15.7 for green, and 0.5 to 2.9 for blue. It is obvious that the blue SCs have the best tolerance for the effect of the variation of the thicknesses. Although the color differences for red (green) is up to 11.2 (15.7), the average values of them are around 4.3 (8). Besides, we investigate the variation of the thickness (e.g., − 5% and 5%) for each layer on the Eff of SC, the corresponding Eff shows a small variation at the range from − 0.1% to 0.4% for the RGB SCs. Thus, we can regard that the efficiency of SC is robust against the typical thicknesses deviation of the DBR and ARCs in experiments.

**Fig. 5:**
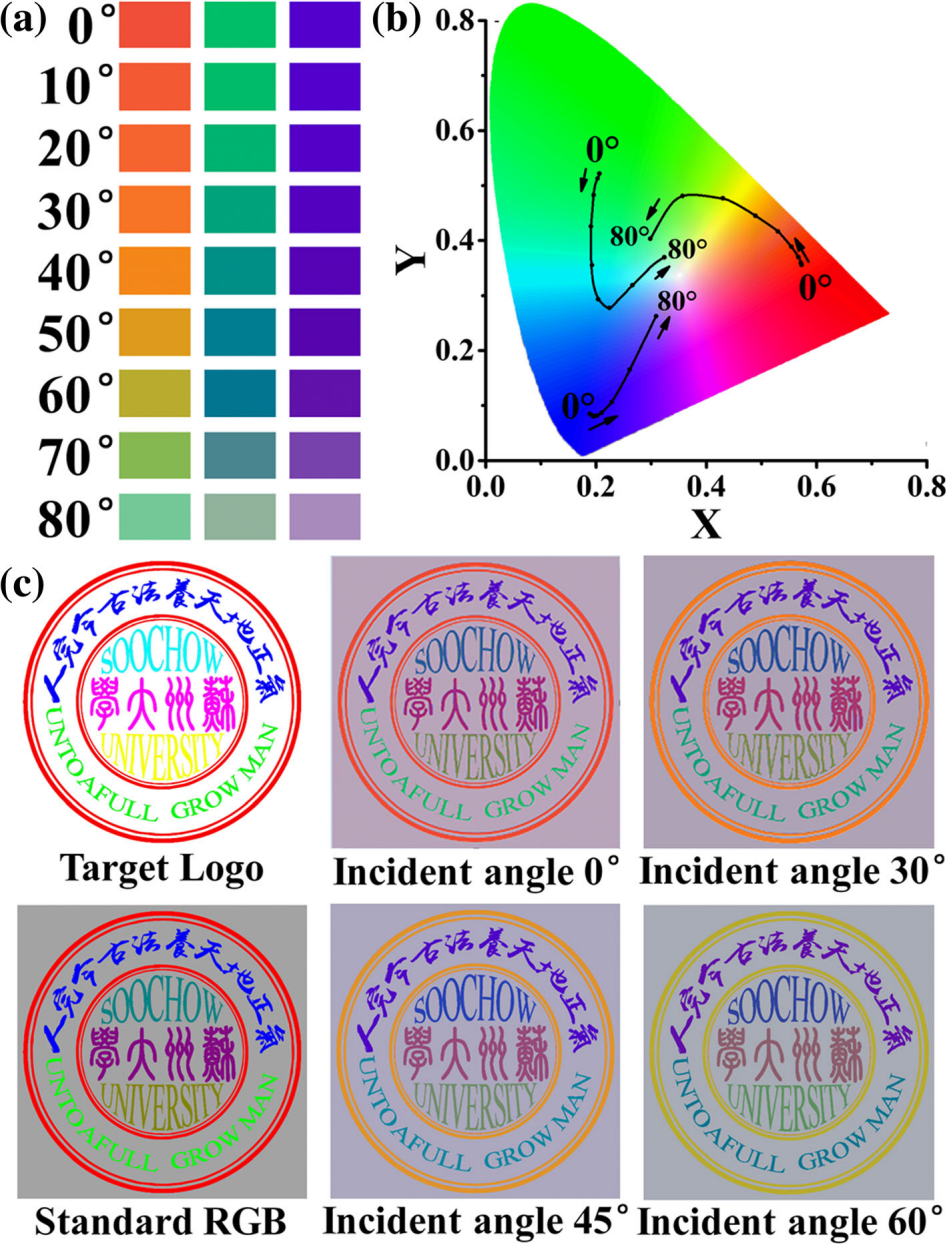
**a** The evolutions of the RGB colors shown by the designed a-Si:H SCs with the incident angle. **b** The variations of the RGB positons in CIE 1931 coordinate with increasing the incident angle. **c** The logo patterns displayed by the a-Si:H SC under various incident angles (0°, 30°, 45°, and 60°). In **c**, the target logo and the logo by standard RGB are included for comparison

Finally, we investigate the effect of the incident angle on the designed colors. Figure 5a exhibits how the designed RGB colors evolve with increasing the incident angle (*θ*). Obviously, the blue and green SCs have better tolerations against the inclined incidence, compared to the red one whose color has been changed from red (*θ* = 0°) to green (*θ* > 70°). For comparison, Fig. 5b shows the loci of the designed RGB colors in the CIE 1931 chromaticity coordinates with continuously increasing *θ*. According to the CIE diagram, the saturation of all colors decreases with increasing *θ*, especially under a large *θ* = 80°, where the colors are very close to the E point (the lowest saturation point). Figure 5c shows the logo of Soochow University under different incident angles. The target logo is composed of seven standard colors, and each color has the most standard hue, saturation, and brightness. The standard RGB is composed of standard red, green, and blue colors with other colors generated from their combinations. Both are used for purpose of comparison. It is clear that the logo is legible even under large incidence angles; however, the pattern colors have been changed to some degree with by increasing incident angle. This leaves room for further optimization in the future.

## Conclusions

In summary, we proposed the thin-film a-Si:H SCs for electricity generation and display application simultaneously for the consideration of new-type BIPVs. The basic RGB display is controlled by the DBRs and the system performance of the color-controlled a-Si:H SC is optimized by applying ARC and buffer layers. The advanced thin-film optical strategies allow the a-Si:H SC to exhibit the high-purity red, green, and blue colors, with the color space comparable to that of sRGB. We further examine the electrical performance based on the optoelectronic model of color-controlled SCs, which show that the power conversion efficiencies can be 4.88%, 5.58%, and 6.54% for R, G, and B cells, respectively. The RGB cells are designed to successfully display the logo of Soochow University, which can be readily distinguished even under a very large incidence angle. Compared to the nanostructured SCs, the proposed a-Si:H SCs in planar configuration can be fabricated by the very mature commercial fabrication processes. Although only a-Si:H SCs are invested, such as the color display principle and electrical evaluation system of color-controlled SCs can be applied for other kinds of SCs. Furthermore, such colored panel can be applied in the modern building walls or roofs to display a pattern, making the esthetic architecture.

## Abbreviations

*A*: Absorption
ARCs: Antireflection coatings
BIPVs: Building-integrated photovoltaics
CAL: Color-adjusting layer
CIE: Commission Internationale de L’Eclairage
DBR: Distributed Bragg reflector
Eff: Photoconversion efficiency
EQE: External-quantum efficiency
FF: Fill factor
F-P: Fabry-Perot
*J*sc: Short-circuit current density
*J*-*V*: Current-voltage
NTSC: National television system commission
PECVD: Plasma-enhanced chemical vapor deposition
*R*: Reflectivity
RCWA: Rigorous coupled-wave analysis
RGB: Red-green-blue
SCs: Solar cells
sRGB: Standard red-green-blue
STCPC: Selectively transparent and conducting photonic crystal
TCO: Transparent conducting oxide
*V*oc: Open-circuit voltage

## References

[1] Laustsen J (2008). Energy efficiency requirements in building codes, energy efficiency policies for new buildings. IEA.

[2] Sartori I, Napolitano A, Voss K (2012). Net zero energy buildings: a consistent definition framework. Energy Buildings.

[3] Henemann A (2008). BIPV: Built-in solar energy. Renew Energ.

[4] Pagliaro M, Ciriminna R, Palmisano G (2010). BIPV: merging the photovoltaic with the construction industry. Prog Photovolt Res Appl.

[5] Yoon JH, Song J, Lee SJ (2011). Practical application of building integrated photovoltaic (BIPV) system using transparent amorphous silicon thin-film PV module. Sol Energy.

[6] Lee KT, Lee JY, Seo S, Guo LJ (2014). Colored ultrathin hybrid photovoltaics with high quantum efficiency. Light: Sci Appl.

[7] Saifullah M, Gwak J, Yun JH (2016). Comprehensive review on material requirements, present status, and future prospects for building-integrated semitransparent photovoltaics (BISTPV). J Mater Chem A.

[8] Lee JY, Lee KT, Seo S, Guo LJ (2014). Decorative power generating panels creating angle insensitive transmissive colors. Sci Rep.

[9] Fukuda M, Lee KT, Lee JY, Guo LJ (2014). Optical simulation of periodic surface texturing on ultrathin amorphous silicon solar cells. IEEE J Photovolt.

[10] Chen YH, Chen CW, Huang ZY, Lin WC, Lin LY, Lin F, Wong KT, Lin HW (2014). Microcavity-embedded, colour-tuneable, transparent organic solar cells. Adv Mater.

[11] Park HJ, Xu T, Lee JY, Ledbetter A, Guo LJ (2011). Photonic color filters integrated with organic solar cells for energy harvesting. ACS Nano.

[12] O’Brien PG, Chutinan A, Mahtani P, Leong K, Ozin GA, Kherani NP (2011). Selectively transparent and conducting photonic crystal rear-contacts for thin-film silicon-based building integrated photovoltaics. Opt Express.

[13] Yang Y, O’Brien PG, Ozin GA, Kherani NP (2013). See-through amorphous silicon solar cells with selectively transparent and conducting photonic crystal back reflectors for building integrated photovoltaics. Appl Phys Lett.

[14] Kuo MY, Hsing JY, Chiu TT, Li CN, Kuo WT, Lay TS, Shih MH (2012). Quantum efficiency enhancement in selectively transparent silicon thin film solar cells by distributed Bragg reflectors. Opt Express.

[15] Dong WJ, Lo NT, Jung GH, Ham J, Lee JL (2016). Efficiency enhancement and angle-dependent color change in see-through organic photovoltaics using distributed Bragg reflectors. Appl Phys Lett.

[16] Lumb PL, Yoon W, Christopher GB, David S, Joseph GT, Robert JW (2013). Modeling and analysis of high-performance, multicolored anti-reflection coatings for solar cells. Opt Express.

[17] Li X, Hylton NP, Giannini V, Lee KH, Ekins-Daukes NJ, Maier SA (2011). Bridging electromagnetic and carrier transport calculations for three-dimensional modelling of plasmonic solar cells. Opt Express.

[18] Li X, Hylton NP, Giannini V, Lee KH, Ekins-Daukes NJ, Maier SA (2013). Multi-dimensional modeling of solar cells with electromagnetic and carrier transport calculations. Prog Photovolt Res Appl.

[19] Shang A, Li X (2017). Photovoltaic devices: opto-electro-thermal physics and modeling. Adv Mater.

[20] Li X, Zhan Y, Wang C (2015). Broadband enhancement of coaxial heterogeneous gallium arsenide single-nanowire solar cells. Prog Photovolt Res Appl.

[21] Shang A, Zhai X, Zhang C, Zhan Y, Wu S, Li X (2015). Nanowire and nanohole silicon solar cells: a thorough optoelectronic evaluation. Prog Photovolt Res Appl.

[22] Chen L, Wu S, Ma D, Shang A, Li X (2018). Optoelectronic modeling of the Si/α-Fe_2_O_3_ heterojunction photoanode. Nano Energy.

[23] Palik E, Ghosh G (1998). Handbook of optical constants of solids.

[24] Das C, Lambertz A, Huepkes J, Reetz W, Finger F (2008). A constructive combination of antireflection and intermediate-reflector layers for a-Si/μc-Si thin film solar cells. Appl Phys Lett.

[25] Kasap SO, Sinha RK (2001). Optoelectronics and photonics: principles and practices.

[26] Volz HG, Simon FT (2001). Industrial color testing.

[27] Kuehni RG (2003). Color chemistry: syntheses, properties, and applications of organic dyes and pigments.

[28] Macleod HA (2010). Thin-film optical filters.

[29] Anderson TH, Faryad M, Mackay TG, Lakhtakia A, Singh R (2016) Combined optical-electrical finite-element simulations of thin-film solar cells with homogeneous and nonhomogeneous intrinsic layers. J Photonics Energy 6 025502

[30] Fujibayashi T, Matsui T, Kondo M (2006). Improvement in quantum efficiency of thin film Si solar cells due to the suppression of optical reflectance at transparent conducting oxide/Si interface by antireflection coating. Appl Phys Lett.

[31] Matsui T, Sai H, Suezaki T, Matsumoto M, Saito K, Yoshida I, Kondo M (2013) Development of highly stable and efficient amorphous silicon based solar cells, Proc. 28th European Photovoltaic Solar Energy Conference, 2213

[32] Lee KT, Jang JY, Zhang J, Yang SM, Park S, Park HJ (2017). Highly efficient colored perovskite solar cells integrated with ultrathin subwavelength plasmonic nanoresonators. Sci Rep.

[33] Cheng HD, Jiang XH, Sun Y, Wang JL (2001). Color image segmentation: advances and prospects. Pattern Recogn.

[34] Wyszecki G, Stiles WS (1982). Color science.

[35] Bube RH (1998). Photovoltaic materials (series on properties of semiconductor materials; Vol. 1).

[36] Rech B, Wagner H (1999). Potential of amorphous silicon for solar cells. Appl Phys A Mater Sci Process.

[37] Kelly PJ, Arnell RD (2000). Magnetron sputtering: a review of recent developments and applications. Vacuum.

